# Ignoring social distancing: advances in understanding multi-species bacterial interactions

**DOI:** 10.12703/r/9-23

**Published:** 2020-12-14

**Authors:** Brittany N Ross, Marvin Whiteley

**Affiliations:** 1 School of Biological Sciences, Georgia Institute of Technology, Atlanta, Georgia, USA; 2 Emory-Children's Cystic Fibrosis Center, Atlanta, Georgia, USA; 3 Center for Microbial Dynamics and Infection, Georgia Institute of Technology, Atlanta, Georgia, USA

**Keywords:** interspecies interaction, multi-species interaction, bacterial environment, bacterial network analysis

## Abstract

Almost every ecosystem on this planet is teeming with microbial communities made of diverse bacterial species. At a reductionist view, many of these bacteria form pairwise interactions, but, as the field of view expands, the neighboring organisms and the abiotic environment can play a crucial role in shaping the interactions between species. Over the years, a strong foundation of knowledge has been built on isolated pairwise interactions between bacteria, but now the field is advancing toward understanding how cohabitating bacteria and natural surroundings affect these interactions. Use of bottom-up approaches, piecing communities together, and top-down approaches that deconstruct communities are providing insight on how different species interact. In this review, we highlight how studies are incorporating more complex communities, mimicking the natural environment, and recurring findings such as the importance of cooperation for stability in harsh environments and the impact of bacteria-induced environmental pH shifts. Additionally, we will discuss how omics are being used as a top-down approach to identify previously unknown interspecies bacterial interactions and the challenges of these types of studies for microbial ecology.

## Introduction

Over the past several decades, it has become clear that microbial interactions have a profound impact in the fields of human health (e.g. oral health^1^), environmental nutrient cycling^2,3^, and bioremediation (i.e. breakdown of industrial waste^4^). The foundation of interspecies interactions research focuses on pairs of species, whose actions have a bidirectional or unidirectional influence on each other^5^. Although there are likely many unknown forms of bacterial interaction, a majority of those described fall within two categories, competitive or cooperative. Competitive interactions occur between species that desire the same nutrients or spatial location. Competing bacteria utilize several mechanisms to outperform their competitor(s), including use of superior resource acquisition mechanisms, rapid nutrient utilization, coordinated social behavior among related individuals, increased motility toward a resource, or impairment/killing of their competitor(s)^6,7^. Cooperation benefits the interacting partners and includes metabolic exchange of a species’ waste or public goods (i.e. cross-feeding and syntrophy), protection, and environmental detoxification^8–10^. More recently, habitat modification has emerged as a major means of competitive and cooperative interactions by shifting environmental parameters (e.g. pH, nutrient concentration) to suboptimal ranges or by stabilizing them^11,12^. Both competition and cooperation can lead to the emergence or decline of species; however, when and where the various types of interaction occur depend on the particular circumstances. In this review, we will focus on the interactions that play a role in the establishment and maintenance of a core functional community as opposed to random interactions that occur outside the context of a community.

In order to investigate how bacterial species in a society interact either physically or chemically, scientific studies must rationally recapitulate some aspect of the community. To date, most interspecies studies have been carried out in laboratory conditions that lack many aspects of the natural environment. These studies have been instrumental in establishing mechanisms of interspecies interactions and the fundamental capacity of bacteria to interact. More recent work has aimed to confirm and expand on these results by using conditions that more closely mimic those of the natural environment^1,12–16^. Using these experimental systems, researchers are revisiting general ecological questions such as the following: how does the surrounding community or physical environment (e.g. nutrients, spatial structure) affect an interaction between two species? At what spatial scale should experiments be carried out? How close do bacteria have to be to interact? And can omics methods tell us which bacteria are interacting? These questions have not fully been answered, but the studies detailed in this review are beginning to shed light on the emerging methods and trends in the study of diverse communities and dynamic environments.

## Mimicking nature’s complexity

Having gained considerable insight into how different species interact, the next question to ask is how these interactions are affected in a native environment. To more closely mimic natural habitats, scientists are adding naturally cohabitating bacteria and using media that resemble the bacteria’s native habitats. Incorporating these techniques has shown that both “who” and “what” are around can impact the extent of bacterial relations.

### There is bacteria all around

To date, most methods of community incorporation have relied on sequencing studies^14,17,18^ to identify commonly co-occurring organisms that are then selectively added to generate a smaller community called a “microcosm”. Community member selections may be biased by ease, interest, scale^19^, inability to culture community members^20^, exclusion of low-abundance but high-impact bacteria^21^, or misleading sequencing data^22^. To address the issue of biased data, several initiatives have generated standardized controls for metagenomic studies, which we support for the validation of existing and new protocols. An alternative to bottom-up methods, which piece together a microbial community, are systems using membrane-separated compartments^15,23^ or dialysis tubing^24^ that allow for experiments to be carried out with the natural consortia. Regardless of the community incorporation technique used, there are several implications of having additional species in an experimental environment. More species increase the possibility for horizontal transfer of genetic materials^25^, altered spatial organization^26^, overall habitat modification^27^, and even altered evolutionary trajectories^28^. Additionally, not only are bacterial species being added but so are the bacteriophages they carry and the membrane vesicles they generate^29,30^. These changes all can affect interactions, which has led researchers to examine if and when pairwise interactions are scalable.

**Figure 1.**
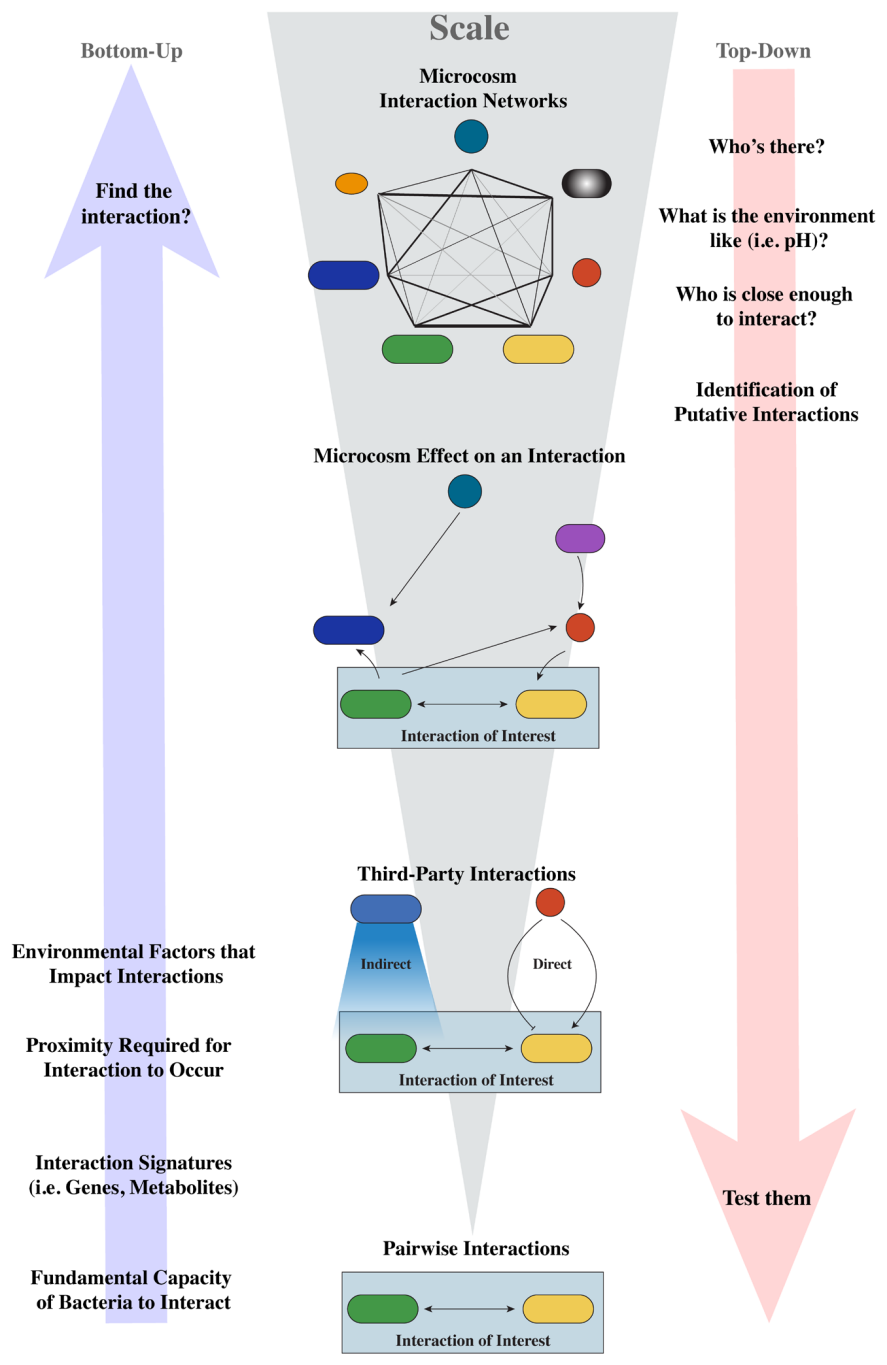
Interspecies interaction knowledge fuels bidirectional studies across scales.

Microcosm experiments have found that individual pairwise interactions are often consistent across community complexity, indicating that bottom-up approaches incorporating progressively more community members are largely, but not always, valid^12–14,18^ (Figure 1). Three-organism experiments are shedding light on why some interactions are not scalable. Addition of a third-party organism to a two-membered community has shown that 1) the strength of pairwise interactions can be affected^13,31–33^, 2) the interactions can be modified to form a multi-way relationship^31,32^, and 3) environmental changes indirectly change bacterial behavior or the abundance of participating species^13,14,33^. Because the presence and strength of an interaction can have a broad effect on a community, studies using a complex community provide insight on the importance of an interaction in an ecosystem. This does not mean pairwise interaction studies are less valuable because they allow us to understand the fundamental capacity of bacteria to interact. Pairwise studies also aid in identifying features such as the genes responsible for interactions, metabolites made or consumed, and proximity requirements for an interaction. The knowledge gained in pairwise interaction can then be leveraged in bottom-up studies to interrogate the role in complex communities. Reciprocally, the increased use of more complex studies is advancing our knowledge of bacterial interaction networks that can fuel new avenues of investigation to be studied at smaller scales (Figure 1).

### The environment impacts bacterial interaction

Unlike multicellular organisms, which have mechanisms to maintain their homeostasis in a changing environment, microbe physiology is more context specific and so are their interactions^4,34–36^. Many studies have found that the media used in an experiment affects interactions^13,33,37^, highlighting the need to recapitulate not only the community but also the abiotic environment. Furthermore, using transcriptomes, several studies have shown that bacterial behavior in the laboratory is not consistent with the behavior in a natural *in situ* environment^38,39^*.* Aiming to more accurately replicate native environments, several new model systems have been developed, including artificial urine media^40^ for an investigation into polymicrobial urinary tract infections, complex skin organoids for skin-community colonization^41^, the inclusion of food-grade fibers in a gut model^42^, as well as several systems facilitating cultivation in native environments^15,16^. These models, based on researcher rationale, are likely superior models, but without validation it is unclear how they compare to the native environment. Studies comparing bacterial gene expression in a model to environmental samples offer an opportunity to iteratively improve model components like nutrient levels, pH, and amino acid presence, allowing for more accurate recapitulation of interactions that occur in nature^39,43,44^. This point also highlights a lack of rationale for the use of general laboratory media when trying to draw conclusions about the natural environment. Media selection justification should not be a biased topic and not restricted to new models. Often studies focused on basic bacterial physiology do not provide justification even if the media was rationally selected for a defined reason (i.e. to support fast growth). We employ that justification for media selection, and the limitations introduced by media should be clearly stated, especially if they are not chemically consistent (e.g. contains yeast extract or tryptone which vary between batches).

Environmental components are important to consider because both nutrient level^13^ and the type of nutrients present^4^ can play a large role in dictating social interactions. In some examples, increased nutrient availability strongly promoted bacterial competition, causing changes in the overall biodiversity^13,37^. When an eight-species soil community was given high levels of nutrients, extensive growth led to more environmental modification, increased negative interspecies interactions, and loss of biodiversity in the community^13^. Similar results were found with a four-species community used in bioremediation. These microbes showed only positive or neutral interactions in a stressful environment; however, upon nutrient addition or reduction of toxicity, competition increased^37^. The correlation between stress and cooperation is consistent with the stress-gradient hypothesis originally described in 1997 noting that “positive interactions may be common, predictable, and pervasive forces in natural communities and in physically harsh environments in particular”^45^. Regardless of whether an interaction is cooperative or competitive, most of the known forms of interactions are mediated through environmental chemical modification by bacteria and are dependent on the environment used in an experiment.

## Habitat modification is a growing field

In bacterial ecology, there is a growing interest in the milieu of a community. Along with the long-standing study of cross-feeding, pH is gaining interest as more studies find that it plays a major role in community composition and stability. Both metabolite usage and pH range are increasingly becoming reliable predictors of interactions, as we will discuss in this section.

### Feeding your neighbors

Nutrient cross-feeding refers to the catabolism of a bacterium’s secreted products by another bacterium. Microbial cross-feeding is a widespread phenomenon^46^ that can alter community composition^17^, structure^4,26^, evolution^47,48^, virulence^49,50^, and antibiotic susceptibility^27,51,52^. Because cross-feeding often fulfills a metabolic requirement, genomic data are increasingly being used to identify the basic metabolic needs of a species and to predict social interactions^33,53,54^. For example, co-abundance and metabolic requirements were extrapolated to reveal that oral interactions were predominately cooperative^53^. One issue faced with predictive models is the lack of knowledge on metabolite preference in communities and hierarchal use of nutrients, which is addressed by Bajic and Sanchez^55^.

Aside from the growing use in predictive models, cross-feeding has been shown to promote community member survival under antibiotic exposure^27,51^. Cross-feeding also leads to the death of community members when metabolic dependencies are present and cooperative interactions are lost owing to antibiotic killing of the most susceptible member^51,56^. Consistent with the findings detailed above showing cooperative interactions in harsh environments, a study showed that cross-feeding led to stabilization of the species when in low-nutrient environments *in vitro* and in the gnotobiotic mouse gut^57^. Together, these findings highlight a recurrent paradigm that low nutrients can promote cooperative interspecies cross-feeding, resulting in community stabilization. Upon the addition of nutrients, some cooperative relationships are lost, and the community composition changes based on fitness levels and environmental modification (i.e. pH). In these events, it is likely that not all cross-feeding interactions are lost unless all metabolic requirements are alleviated or organisms change their metabolic preference upon the addition of nutrients.

### The impact of pH

The role of environmental pH shifts is a prominent topic in the study of interspecies interactions. The summation of pH changes by the community dictates the fate of community members^33^. For example, opposing pH shifts by bacteria can be considered a positive interaction because the resulting pH stabilization promotes community synergy^11^, while pH manipulation by a transient invader can influence the stable state of a competing two-member system^12^. Shifts in pH can also modulate the antimicrobial tolerance of cohabitating species^27^. Environmental pH can affect not only the bacteria but also chemicals as well. It is still unclear if the change in the chemical affects chemical–microbe interaction or the interaction is a result of the change in bacterial behavior alone.

Outside of the laboratory, pH has been shown to be a major driver of community composition^58^ and diversity^59^ in natural soil samples. One of the many explanations for the strong role of pH is that it affects energy yields of microbial respiration, giving rise to pH limitations on community membership^60^. Using a species’ metabolic properties, pH preferences, and strength of environmental modifications, interspecies interactions, community membership, and community stability have been shown to be predictable^33^. The next question is if other factors such as oxygen, metabolite concentrations, or broad environmental parameters like precipitation/wetness in soil communities can just as easily be predictable parameters for community composition and interspecies interaction. Additionally, a parameter that should be strongly considered in future studies is the scale at which these parameters are predictive, as interactions can vary across the spatial-temporal landscape.

## Spatial structure

A long-standing question is how close bacteria have to be to interact. Overall, the literature shows that interactions occur in short ranges with neighbors^61,62^. Some types of interactions are intimate and require cell-to-cell contact, while diffusible chemicals have larger ranges, which increase based on their physiochemical properties (e.g. volatility). A possible mechanism of long-range interaction is extracellular electron transfer, where bacteria can donate or accept electrons from the environment at distances up to 1 cm via surface-attached pili or environmental conductive materials^63,64^. Extracellular electron transfer may serve as a novel mechanism of interspecies interaction; however, more work is needed in this area of research. Using the information gained on distance requirements for specific types of interactions, mechanisms of interspecies interactions can be speculated by knowing the average distance between two species. It is also possible that a ripple effect of multiple interacting species can dictate spatial organization.

Because different types of interaction occur at different scales and distances, the use of multiple techniques is often necessary to investigate interspecies interactions. For example, the use of micro-scale bioprinting of microbial colonies demonstrates that metabolite sharing between species is distance dependent and adding a competitor adjacent to or in the path of metabolite diffusion can curtail metabolite cross-feeding^65^. Perturbations in microsite colonization correlated to changes in metatranscriptomics have been used to identify distinct competition and cooperation interactions occurring during spatial organization that facilitate biomass expansion^26^. Use of electron topography and fluorescence transmission electron microscopy proved that *Clostridium ljungdahlii* and *Clostridium acetobutylicum* in close proximity can undergo interaction that involves cell wall fusion, allowing for large-scale protein and RNA exchange^66^.

As you might expect, adding just one species to a system or altering the environment (i.e. oxygen) can markedly change the community’s spatial architecture likely as a result of adaptation to newly developed niches^67,68^. These studies indicated that model systems can be improved through an iterative process by comparing community organization in the lab to a real-world community. Further insight into natural community arrangement has been afforded by advances in imaging, particularly combinatorial labeling fluorescence *in situ* hybridization techniques coupled with microscopy^69–73^. One of the technical gaps in spatial ecology is connecting visual observations with mechanisms. Two newer avenues that can be used to address this knowledge gap are Raman spectroscopy and imaging mass spectrometry, which allow for visualization of cells and corresponding assessment of the chemical topography at high resolution^69,74^. Additionally, next-generation approaches have been proposed that combine phenotype probing and observation before endpoint assays such as omics to allow for mechanistic insights into the spatial organization of a community^75^.

## The continuing rise of omics

The use of omics techniques has played a critical role in understanding bacterial interactions, and, as the technologies continue to improve and costs decrease, we anticipate these techniques will factor more prominently. The most frequently used omics approaches provide an assessment of who is present in a community (ribosomal RNA gene amplicon sequencing, metagenomics, and metatranscriptomics), what they can do (metagenomics^76^), and what they are doing (metatranscriptomics^34^, metaproteomics^4^, and metabolomics^67^). Beyond these techniques, transposon-insertion sequencing (Tn-seq) has been shown to be a high-throughput method to study interactions between species^77,78^. When carried out on wound and oral communities, Tn-seq found that previously non-essential genes become essential when an organism is co-cultured with another species^77,78^.

**Figure 2.**
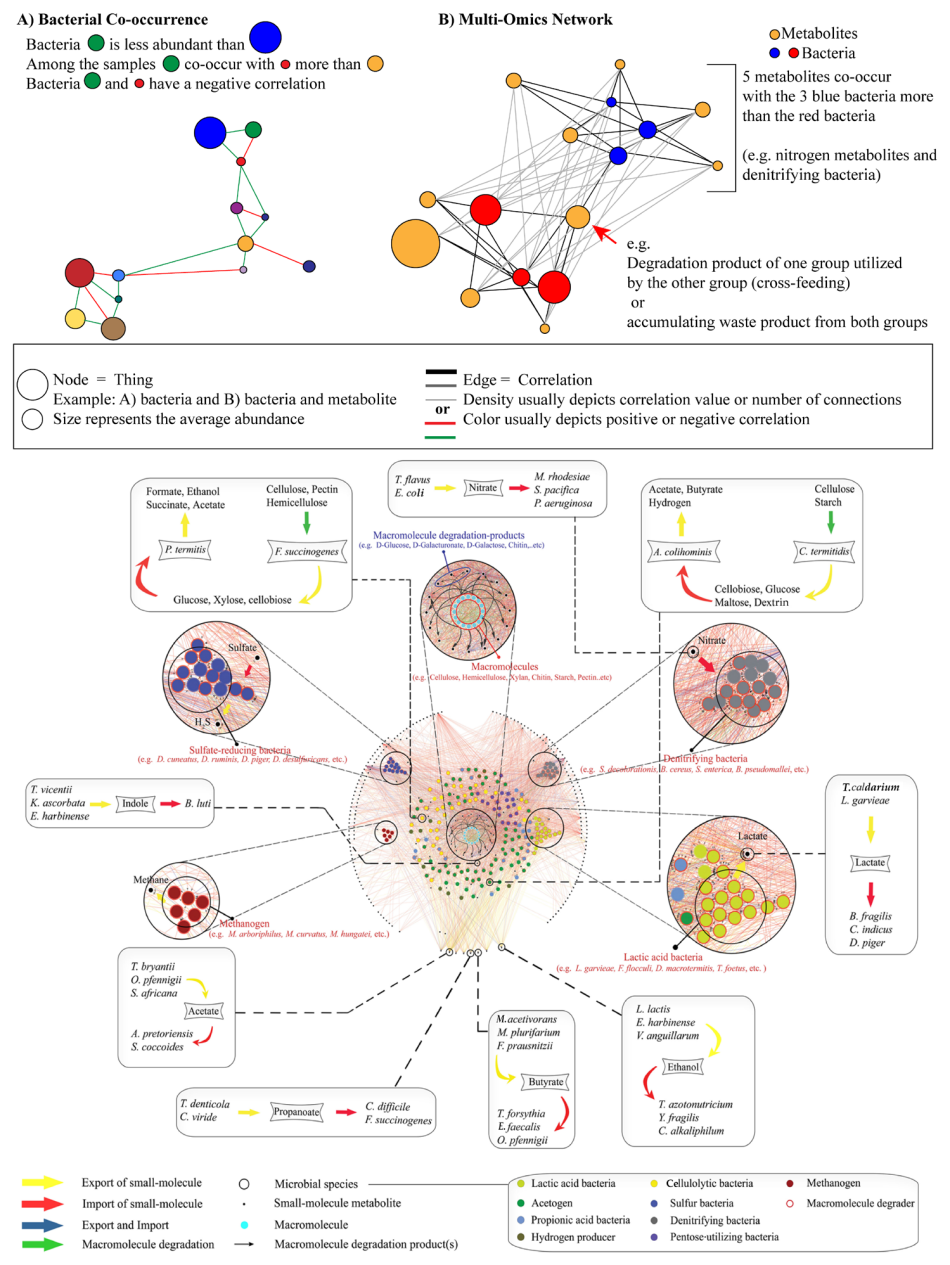
Flavors of network analyses used to infer bacteria interaction. Depending on the analysis, (**A**) one or (**B**) more types of nodes may be incorporated (i.e. bacteria, metabolites, genes, etc.). Edges represent the correlation, which is calculated between each node. (**A**) Often a threshold is used, and only significant co-occurrences are made visible to reduce complexity. Alternatively, all edges are shown (**B**), but with more complex networks the image becomes hard to interpret. (**C**) To illustrate the complexity and information gained from a network, work by Sung *et al*. is provided showing species abundance, metabolite export, and import^80^. This figure incorporates known degradation reactions and utilization with co-occurring microbes to identify interactions between species as they digest lignocellulose in the termite gut (reprinted under the Creative Commons Attribution 4.0 International License)^81^. *Amycolatopsis pretoriensis*, *Anaerotruncus colihominis*, *Bacillus cereus*, *Bacteroides fragilis*, *Bacteroides luti*, *Burkholderia pseudomallei*, *Calidifontibacter indicus*, *Chitinispirillum alkaliphilum*, *Clostridium difficile*, *Clostridium termitidis*, *Cnodalon viride*, *Desulfotomaculum ruminis*, *Desulfovibrio cuneatus*, *Desulfovibrio desulfuricans*, *Desulfovibrio piger*, *Dysgonomonas macrotermitis*, *Ethanoligenens harbinense*, *Escherichia coli*, *Enterococcus faecalis*, *Faecalibacterium prausnitzii*, *Fibrobacter succinogenes*, *Flexilinea flocculi*, *Kluyvera ascorbata*, *Lactococcus garvieae*, *Lactococcus lactis*, *Mesorhizobium plurifarium*, *Methanobrevibacter arboriphilus*, *Methanobrevibacter curvatus*, *Methanosarcina acetivorans*, *Methanospirillum hungatei*, *Mycobacterium rhodesiae*, *Oxobacter pfennigii*, *Pilibacter termitis*, *Pseudomonas aeruginosa*, *Salinispora pacifica*, *Salmonella enterica*, *Shewanella decolorationis*, *Spirochaeta africana*, *Spirochaeta coccoides*, *Tannerella forsythia*, *Tessaracoccus flavus*, *Treponema azotonutricium*, *Treponema bryantii*, *Treponema caldarium, Treponema denticola*, *Treponema vicentii*, *Tritrichomonas foetus*, *Vibrio anguillarum*, *Youngiibacter fragilis*.

The use of omics has been particularly helpful in complex communities where multiple interactions are taking place. As a top-down method, omics are being used to infer interactions between species through network analyses. Often used for human social and protein interactions, network methods rely on the probability of bacterial species co-occurring to infer cooperation via positive correlation and competition via negative correlation^40,53^ (Figure 2A). Network analysis is also being expanded to include metabolite transport and usage^79–81^ or gene expression from multiple species^82^ to aid in mechanism identification (Figure 2B). Although these methods are proving to be very useful, more work is needed to address varied accuracy between available data analysis tools, difficulty inferring some interaction types, particularly complex oscillating interactions (e.g. predator versus prey), and limited use for low-abundance organisms that have a strong impact in the community^21,83^. Owing to the correlative nature of network interaction, the complexity and non-linear dynamics of communities inferring interactions from covariance should be viewed with skepticism until coupling with empirical data to prove interactions (i.e. colocalization, stable isotope labeling, and co-culturing assays)^84,85^. This is illustrated by recent papers incorporating known metabolite transport, usage, and production with species co-occurrence into a network and finding both known and yet-to-be-confirmed interactions (Figure 2C)^80,81^. Although there is skepticism in interpreting novel network data and necessary improvements in microbial ecology analysis techniques are necessary, network methodologies are allowing scientists to better understand complex multispecies communities.

More frequently, studies are using a multi-omics approach that incorporates data on factors such as bacterial abundance, transcriptomics, proteomics, or metabolomics to elucidate multi-species interaction networks and the mechanisms of interactions^4,76^. For example, metagenomics, metatranscriptomics, and targeted metabolite analysis led to the identification of interspecies interactions involved in the environmental biodegradation of bisphenol A^76^. Combinations of multi-omics approaches and experimental validation are paving the way for the next generation of interspecies interaction discoveries.

## Discussion

Similar to other fields in biological sciences, adapting experiments to mimic the natural world is gaining traction in microbial ecology. Approaches mimicking complex bacterial habitats are showing that cohabitants and environmental factors affect bacterial interactions; thus, experimental data interpretation is limited by study design. By working with more complex communities, more interspecies interactions are being included, making it difficult to untangle the network of actions and reactions by different species. The data that can be gleaned based on scale is a pertinent topic because the outcomes of a single interaction can lead to larger consequences such as microbiome dysbiosis and diseases such as gingivitis^86^, gastrointestinal diseases, and obesity^87^. The scale used in a study dictates the inclusion of the community, the abiotic environment, and what questions can be asked^19,88^. Several studies show that bottom-up approaches are useful to answer many questions^12–14,18^; however, they have several limitations and therefore community studies are needed before assuming that a pairwise interaction occurs across scales^13^. Reciprocally, top-down methods that rely on omics lack conclusive proof of an interaction and must be validated by small-scale studies. Likewise, the environmental system can impact interactions^4,34–36,38,39,43^; therefore, new model systems need to be validated^39,43,89^ and old model systems need to be rationalized if used.

Investigations of bacterial interactions at the community level have highlighted the importance of cooperative interactions and the role of environmental nutrients in influencing interactions and community stability. Competitive interactions are more often studied, but hopefully these recent findings will inspire more interest in cooperative mechanisms between species. Additionally, as more relevant and sophisticated experiments are performed, new questions arise, including the following: can metabolite concentrations or oxygen levels be used as predictable parameters of interspecies interactions? Within a community, what are the distinct niches and what interactions govern niche establishment? Many of these questions will likely be answered with the help of omics-level data analysis coupled with carefully designed experimental systems. A significant challenge is still tracking the origin of interaction, particularly with chemical signatures. Progress in metabolomics offers more advanced and holistic methods for examining environmental modification and cross-feeding, including mass spectroscopy imaging and Raman methods, which have the potential to begin to indicate metabolite origins.

Intercalation of data from multiple omics datasets is providing an increasingly granular view of known interactions and is identifying unknown community-based interspecies interactions for further study. With this new frontier, appropriate data use is critical, and analysis will often need collaboration between subject matter experts, modelers, bioinformaticians, and computer scientists. Omics data can be leveraged not only by the originating lab but also by others, yet one out of every five metagenomic studies since 2016 has not been deposited into a repository^90^. The lack of commitment for open access omics data is stifling progress, and so we want to close by stressing the importance of making raw omics data open access for the betterment of the field.
